# Comparative analyses of physicochemical and volatile flavor characteristics in hooked, trawl‐net, and radar‐net hairtail (*Trichiurus haumela*) muscles during long‐term cryopreservation at −18°C

**DOI:** 10.1002/fsn3.4381

**Published:** 2024-08-23

**Authors:** Shanshan Shui, Yu Chen, Hongbo Yan, Jia Song, Shucheng Liu, Soottawat Benjakul, Bin Zhang

**Affiliations:** ^1^ College of Food Science and Pharmacy Zhejiang Ocean University Zhoushan China; ^2^ Pisa Marine Graduate School Zhejiang Ocean University Zhoushan China; ^3^ College of Food Science and Technology Guangdong Ocean University Zhanjiang China; ^4^ International Center of Excellence in Seafood Science and Innovation, Faculty of Agro‐Industry Prince of Songkla University Hat Yai Thailand

**Keywords:** cryopreservation, fishing method, hairtail, microstructural, physicochemical, quality characteristics, volatile flavor

## Abstract

Chemical analysis showed that pH, b* values, myosin turbidity, carbonyl content, and surface hydrophobicity elevated in hooked, trawl‐net, and radar‐net hairtail (*Trichiurus haumela*, HH, TH, and RH) muscles with the prolonged cryopreservation time (‐18℃, 120 d). In contrast, L*, a* values, textural properties, and myosin solubility existed decreasing trends. Microstructural results showed that freezing resulted in disordered myofibrils, decreased collagen fibers, widened myofibrillar space, and increased fragmentation in hairtail muscles. Furthermore, volatile flavor analysis suggested that aldehydes, ketones, alcohols, and amines were the key factors for the overall flavor formation in hairtails during cold storage. Pearson correlation coefficient analysis revealed that the color, texture, and protein oxidation had close correlations with VOCs. Among the three different kinds of hairtail, fresh RH fillets exhibited an attractive aroma with high economic value, long‐term frozen TH muscle tissues were prone to deterioration in texture, microstructure, and flavor, and the HH samples presented stable quality characteristics and storage performance.

## Graphical abstract

Muscle quality of three hairtail species were investigated during frozen storage. VOCs were identified in three hairtail species during frozen storage Fresh RH samples exhibited an attractive aroma with high economic value.

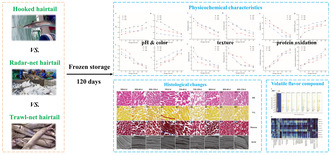

## 1 INTRODUCTION

Hairtail (*Trichiurus haumela*), an important Marine fishing resource in China, with a total production of 903,498 tons in 2022 (Fishery Bureau, 2023), is broadly distributed in the Indian Ocean, Atlantic Ocean, and Eastern Pacific Ocean (Liu et al., 2021). According to the different fishing methods and living areas, hooked hairtail (fished with a hook and line; living in the deep ocean offshore), trawl‐net hairtail (fished with a trawl net; living in the epipelagic ocean of open seas), and radar‐net hairtail (fished with a radar net; living in the deep ocean of offshore), all belonging to the *Trichiurus haumela*, are the three most common kinds of hairtail in Zhejiang Province, China. Hairtail is popular with consumers for its delicious taste and is rich in protein, polyunsaturated fatty acids, minerals, vitamins, and essential amino acids (Koddy et al., 2021). However, hairtail is prone to perishable during capture, transportation, storage, processing, and marketing, mainly ascribed to the high level of unsaturated fatty acids, high endogenous enzyme activity, and microbial reproduction and contamination (Hu et al., 2021).

Frozen storage has been commonly used as a method of hairtail preservation to sustain the quality characteristics and lengthen the shelf‐life of hairtail (Yan et al., 2022). Several studies have demonstrated that the quality characteristics of muscle tissues in hairtail exhibit significant variation during cryopreservation, including a reduction in water activity; textural properties, e.g., hardness, springiness and chewiness; myofibrillar Ca^2+^‐ATPase activity and active sulfhydryl content; an increase in levels of peroxide value and thiobarbituric acid reactive substances; and the destruction of microstructure by ice crystallization (Fang et al., 2022; Luan et al., 2018; Shui et al., 2022). These changes cause undesired texture and flavor, tissue disarrangement, and lipid and protein oxidation.

Both protein oxidation and lipid oxidation can occur during frozen storage, and these two processes are interrelated. The byproducts of lipid oxidation, such as aldehydes and peroxides, can react with proteins, leading to protein oxidation. This process can cause protein cross‐linking, aggregation, and denaturation, affecting the functionality and nutritional value of the proteins (Zhan et al., 2022). According to our knowledge, most studies focus on the muscle quality characteristics in the single kind of hairtail; a comprehensive systematic investigation has yet to be performed to compare the similarities and differences of muscle protein and lipid stability in different kinds of hairtail under cold stress. Therefore, this current study aimed to investigate and compare the physicochemical, myosin functional properties, tissue microstructure, and volatile flavor properties of muscle in hooked hairtail (HH), trawl‐net hairtail (TH), and radar‐net hairtail (RH) during 120 days of long‐term frozen storage, which defines a theoretical foundation database for the variation of quality characteristics in different kinds of hairtail during storage and processing.

## 2 MATERIAL AND METHODS

### 2.1 Chemical reagents

Hematoxylin, eosin, Van Gieson, and Masson's trichrome were obtained from Promega Biotech Co., Ltd. (Beijing, China). All other reagents were analytically pure and obtained from Sinopharm Chemical Reagent Co. Ltd. (Shanghai, China).

### 2.2 Hairtail samples and treatments

Fresh HH, TH, and RH (*T. haumela*), weighing 500–600 g, were acquired from the Zhoushan International Aquatic Products Market (Zhoushan, China). TH had a long body and large eyes; HH and RH had smaller body lengths and small eyes; and the skin of RH was slightly damaged. The acquired hairtail samples were packed into an ice case and returned to the laboratory. Arrived at the destination, the hairtail was washed under cold running water and removed the head and guts. Next, the hairtail samples were cut into 5–6 cm blocks, and every 3 blocks were put into a ziplock polyethylene bag. Lastly, the processed hairtail samples were cryopreserved for 120 days (−18°C) and sampled every 20 days. The hairtail batches were pre‐thawed at 4°C for 3 h before the following indicators were tested in triplicate (*n* = 3).

### 2.3 pH and color analysis

The pH value of hairtail batches was performed as reported by Lin et al. (2021) with some modifications. Approximately 3 g of hairtail fillets were added with 9 times (w/v) 0.09% saline solution and then homogenized at 12,000 × *g* for 1 min (FJ200‐S, Shanghai Lichen‐Bangxi Instrument Technology Co., Ltd., Shanghai, China). After standing at room temperature for 20 min, 10 mL of supernatant was acquired to determine the pH value with a pH apparatus (PHS‐3C, Shanghai INESA Scientific Instrument Co., Ltd., Shanghai, China).

The color of hairtail samples was determined, as reported by Jiao et al. (2022). The surface of hairtail fillets was selected for color measurements by a CR‐10 portable chromometer (Konica Minolta Holdings, Inc., Osaka, Japan). The color was represented by the L* index (brightness), a* index (−a, greenness; +a, redness), and b* index (−b, blueness; +b, yellowness).

### 2.4 Textural properties analysis

The texture properties of hairtail samples were conducted, as reported by Zhu et al. (Zhu et al., 2022). The springiness (mm), chewiness (mJ), adhesiveness (N), and shearing force (N) of hairtail fillets were carried out by a food texture profiler (TMS‐Pro, Food Technology, Inc., VA, USA) prepared with a P36/R probe. The test and post‐test rates were set as 60 mm/min with a return height of 25 mm and a compression ratio of 60%.

### 2.5 Protein oxidation analysis

Myosin extracted from dorsal muscles in HH, TH, and RH were prepared, referring to the mean given by Wang et al. (Wang et al., 2022). The concentration of extracted myosin was measured according to the Bradford assay (Kruger, 2009) utilizing a UV‐2600A spectrophotometer (Unico (Shanghai) Instrument Co., Ltd., Shanghai, China). Finally, the myosin solution was deliquated to 1 mg/mL with Tris–HCl buffer for the following analyses.

The solubility of myosin was measured by referring to the mean given by Lv et al. (2022). The extracted myosin solution (1 mg/mL) was centrifuged (CF16RN, Hitachi, Ltd., Tokyo, Japan) at 5000 × *g* (4°C) for 15 min to determine the protein concentration of the supernatant. Myosin solubility was examined by dividing the concentration measured above by a percentage of 1 mg/mL.

The turbidity of myosin was obtained by estimating the absorbance at 340 nm according to the method reported by Shui, Qi, et al. (2021a) with minor modifications.

The carbonyl level of myosin was determined by reacting with DNPH, as reported by Zhang et al. (2020). The carbonyl level was detected at an absorbance of 365 nm and expressed as nmol carbonyl per mg myosin.

The surface hydrophobicity of myosin was evaluated utilizing BPB as given by Lv et al. (2022). The surface hydrophobicity was detected at an absorbance of 595 nm and expressed as BPB bound per μg.

### 2.6 Histological changes analyses

Hematoxylin and eosin (H&E) staining, Van Gieson (VG) staining, Masson staining, and scanning electron microscopy (SEM) analyses were adopted to analyze and compare the variations of muscle structure in HH, TH, and RH during 0, 60, and 120 days of frozen storage.

The H&E, VG, and Masson staining were conducted as described by Shui et al. (Shui, Yao, et al., 2021b; Zhang et al., 2020) and Zhu et al. (2022). Briefly, after fixation, dehydration, embedding, and slicing of hairtail muscle tissue, staining was performed using H&E, VG, and Masson dye, respectively. Images of hairtail samples were photographed using a light microscope (BX51, Olympus, Japan) under 400× magnification.

The SEM analysis was determined after fixation, dehydration, immersion, drying, and coating of hairtail muscle tissue (Zhu et al., 2022). Images of hairtail samples were photographed with an electron microscope (JSM‐6390LVJ, EOL Ltd., Tokyo, Japan) under 200 × magnification.

### 2.7 Volatile flavor compound analysis

Volatile flavor compounds of hairtail muscles were conducted referring to the method given by Du et al. (2022) with some modifications. Muscle tissues (2 g) were put into a 20 mL headspace vial and cultivated at 90°C for 15 min. Five‐hundred microliter of headspace gas was detected using the gas chromatography‐ion migration spectrometry (GC‐IMS) technology by a FlavourSpec® flavor analyzer (G.A.S., Inc., Dortmund, Germany). The volatile organic compounds (VOCs) were recognized and quantified by matching the retention index, migration time, and peak intensity in the GC‐IMS database.

### 2.8 Data analysis

Three parallel replicates were measured in each hairtail sample (*n* = 3). All figures were portrayed utilizing Origin 2018 software (OriginLab Corporation, Northampton, USA). All data were shown as the mean ± SD and were detected utilizing statistical analysis software (SPSS Inc., Chicago, USA). The significant differences between frozen storage time and kinds of hairtail were conducted using Duncan's multiple range test (*p* < .05), as shown in Table S1.

## 3 RESULTS AND DISCUSSION

### 3.1 PH analysis

**FIGURE 1:**
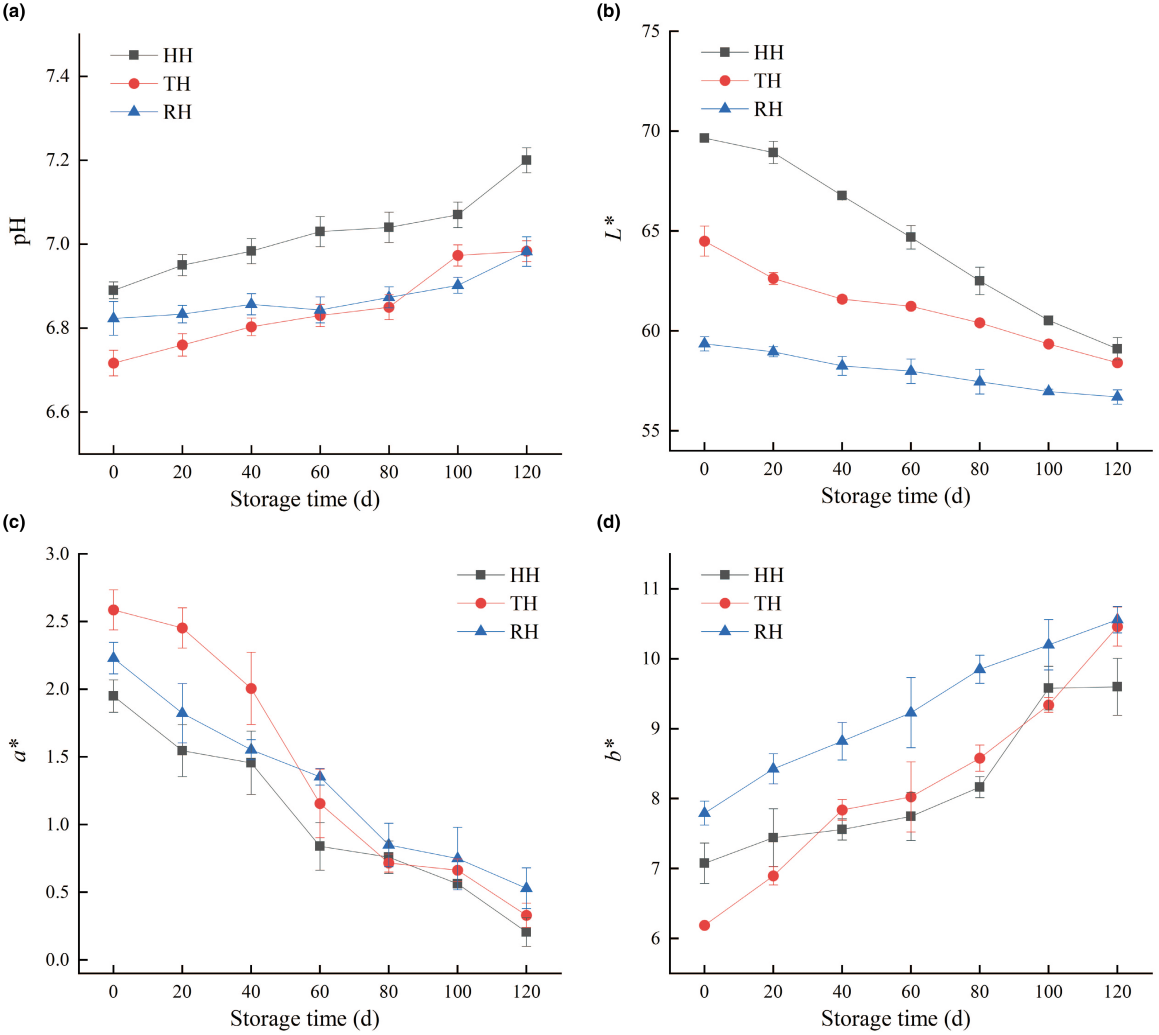
Changes in pH (a), L* (b), a* (c), and b* (d) of hooked hairtail (HH), trawl‐net hairtail (TH), and radar‐net hairtail (RH) muscles during frozen storage.

The pH value is closely connected with the freshness and storage conditions of aquatic products, the post‐mortem limit of which for consumer acceptability is usually below 7.0 (Zhang et al., 2012). As shown in Figure 1a, the pH values of fresh HH, TH, and RH samples were measured in the range of 6.71–6.89, which gradually increased to 6.98–7.20 on 120 d of cryopreservation. This increment could be due to the decomposition of nitrogen fraction and the amassing of alkaline compounds in muscle, such as ammonia, indole, dimethylamine, and trimethylamine, primarily derived from microbial spoilage and activated endogenous enzymes (Shiekh et al., 2021). Compared with HH muscle, TH and RH muscles presented lower pH values during the whole storage period. TH and RH catching in fishing nets struggled violently, easily decomposing glycogen and producing lactic acid, which was prone to maintaining a relatively lower pH value (Wu et al., 2021).

### 3.2 Color analysis

The color of aquatic products, as the first source for consumers to obtain product information, is one of the main factors affecting consumers' purchasing decisions (Ma et al., 2015). Changes in color profiles (L*, a*, and b*) of HH, TH, and RH muscles during frozen storage were presented in Figure 1b–d, respectively. The L*, a*, and b* values were in the range of 59.36–69.66, 1.95–2.59, and 6.19–7.79 on day 0, respectively, indicating that all the fresh hairtail samples exhibited bright red color. The L* and a* values of all hairtail batches tended to decrease, whereas the b* value significantly increased as storage time increased, which were in accord with the color changes in the large yellow croaker (*Pseudosciaena crocea*) (Jiao et al., 2022). The color variation was closely related to the occurrence of protein and lipid oxidation under cold stress, which was manifested in the amassing products of protein and lipid oxidation, e.g., carbonyls and malonaldehyde (Cropotova et al., 2020). Additionally, myoglobin is the hemoglobin responsible for the color of fish. The autoxidation of iron atoms in the center of the heme group changes the red oxymyoglobin into brown metmyoglobin under cryopreservation of fish muscle, which is attributed to the decreased L* and a* values and the increased b* value (Singh et al., 2021). Notably, the initial HH fillet showed the brightest color with the least redness in comparison with the other fillets, and the fresh RH sample had the lowest brightness with the most yellowness, which was probably due to the different fishing methods. HH adopted relatively mild fishing methods with a hook and line, while RH rubbed with each other during the fishing process, which made it easy to destroy the gloss of the skin.

### 3.3 Textural properties analysis

**FIGURE 2:**
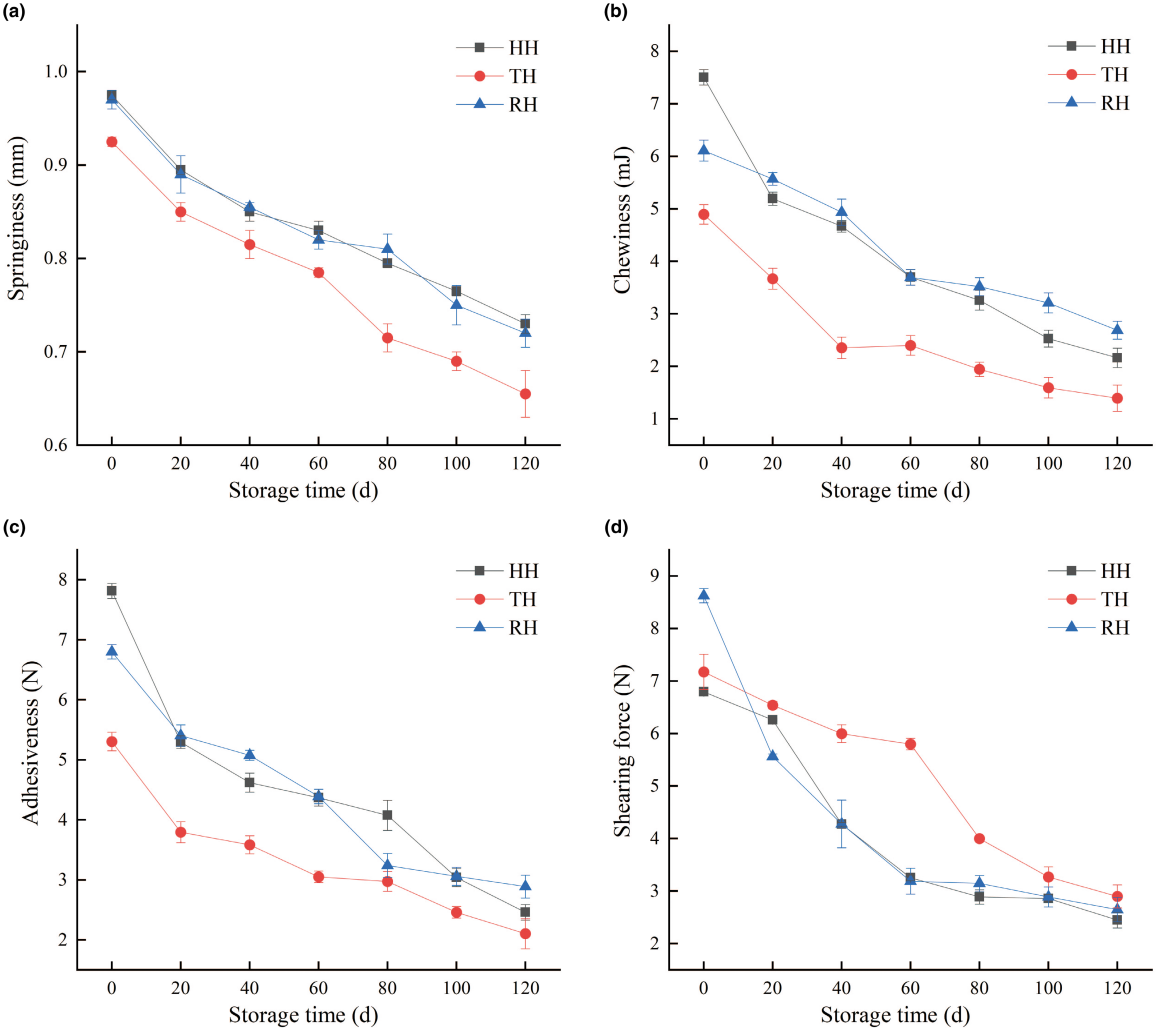
Changes in springiness (a), chewiness (b), adhesiveness (c), and shearing force (d) of hooked hairtail (HH), trawl‐net hairtail (TH), and radar‐net hairtail (RH) muscles during frozen storage.

The textural stability of fish muscle responds to the morphology and structure of myofibrillar proteins, collagen, and connective tissues during transportation, processing, marketing, and storage (Jiao et al., 2022). Similar down‐regulating trends in springiness, chewiness, adhesiveness, and shearing force of three different hairtails were found during 120 d of frozen storage, which experienced a significant decline in the first 20 days and leveled off in the last 20 days (Figure 2). The undesirable texture deterioration might result from the protein oxidation/denaturation inducing the destruction of muscle fibers and myofibrillar proteins, e.g., myosin, actin, and tropomyosin, further to the disorganization of connective structures (Yu et al., 2021). Furthermore, endogenous enzymatic actions (i.e., cathepsin and collagenase) likely hydrolyzed proteins and destroyed the connective tissues, leading to the quick growth and reproduction of microorganisms, promotion of spoilage progress, and a reduction in the texture of fish muscle tissues (Wang et al., 2020). There were no significant differences between HH and RH fillets for all indicators of textural properties. TH muscle tissues presented the lowest springiness, chewiness, and adhesiveness levels during the whole frozen storage period, which was likely closely connected to the living areas of the hairtail. Compared with HH and RH living in the deep ocean, the TH living in the epipelagic ocean had a relatively small range of activity, resulting in poor textural properties.

### 3.4 Protein oxidation analysis

**FIGURE 3:**
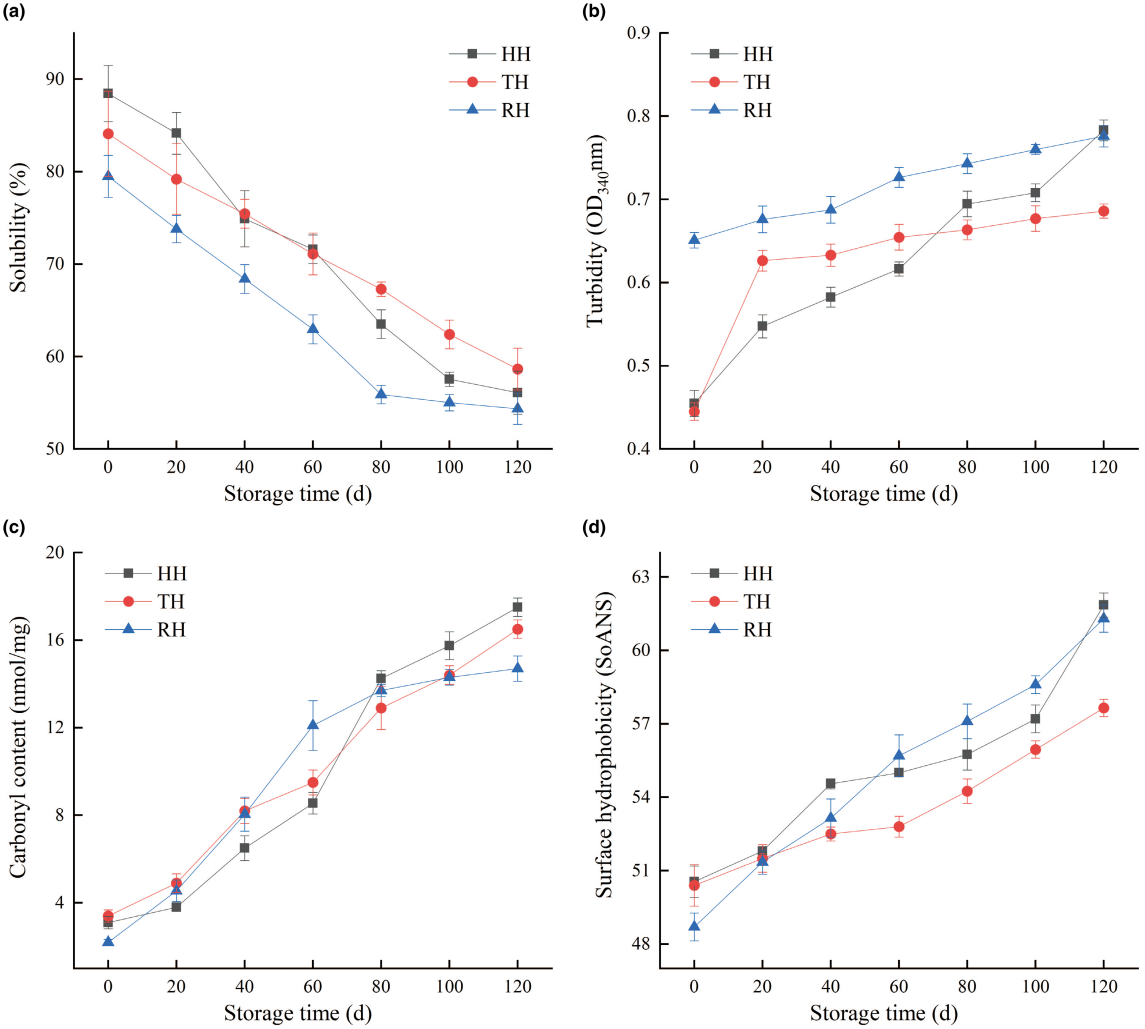
Changes in myosin solubility (a), turbidity (b), carbonyl content (c), and surface hydrophobicity (d) of hooked hairtail (HH), trawl‐net hairtail (TH), and radar‐net hairtail (RH) muscles during frozen storage.

The degree of protein oxidation was measured as the levels of myosin solubility, turbidity, carbonyl content, and surface hydrophobicity in hairtail fillets (Figure 3). A similar decreasing trend of myosin solubility was observed in HH, TH, and RH fillets with prolonged storage time. Whereas myosin turbidity, carbonyl content, and surface hydrophobicity progressively increased during the whole frozen storage period, which was in line with the result of shrimp mud in our previous report (Shui, Qi, et al., 2021a). The relative lower myosin solubility and higher turbidity, carbonyl content, and surface hydrophobicity showed in all muscle tissues at the end of storage, suggesting the occurrence of protein oxidation under long‐term cold stress. The cold conditions stretched and unfolded the peptide backbone, exposed hydrophobic amino acid side chains in myosin, and interacted with reactive oxygen species through non‐covalent and covalent, resulting in the looseness and destabilization of myosin structure (Nyaisaba et al., 2019). The change in myosin structural properties manifested by fragmentation, aggregation (a decrease in solubility and an increase in turbidity), and dysfunctionality of myosin, leading to the upregulation of carbonyl content and surface hydrophobicity. Additionally, protein oxidation was often accompanied by lipid oxidation through radical chain reaction, and the latter could catalyze the occurrence of the former (Xiong et al., 2021). In the current study, the RH sample showed the highest turbidity on day 0, and the TH fillet presented the lowest turbidity and surface hydrophobicity on day 120 of frozen storage, which might be attributed to the specific protein components and their abundance in the muscle tissue of different hairtail (Shui, Yao, et al., 2021b).

### 3.5 Histological changes analyses

**FIGURE 4:**
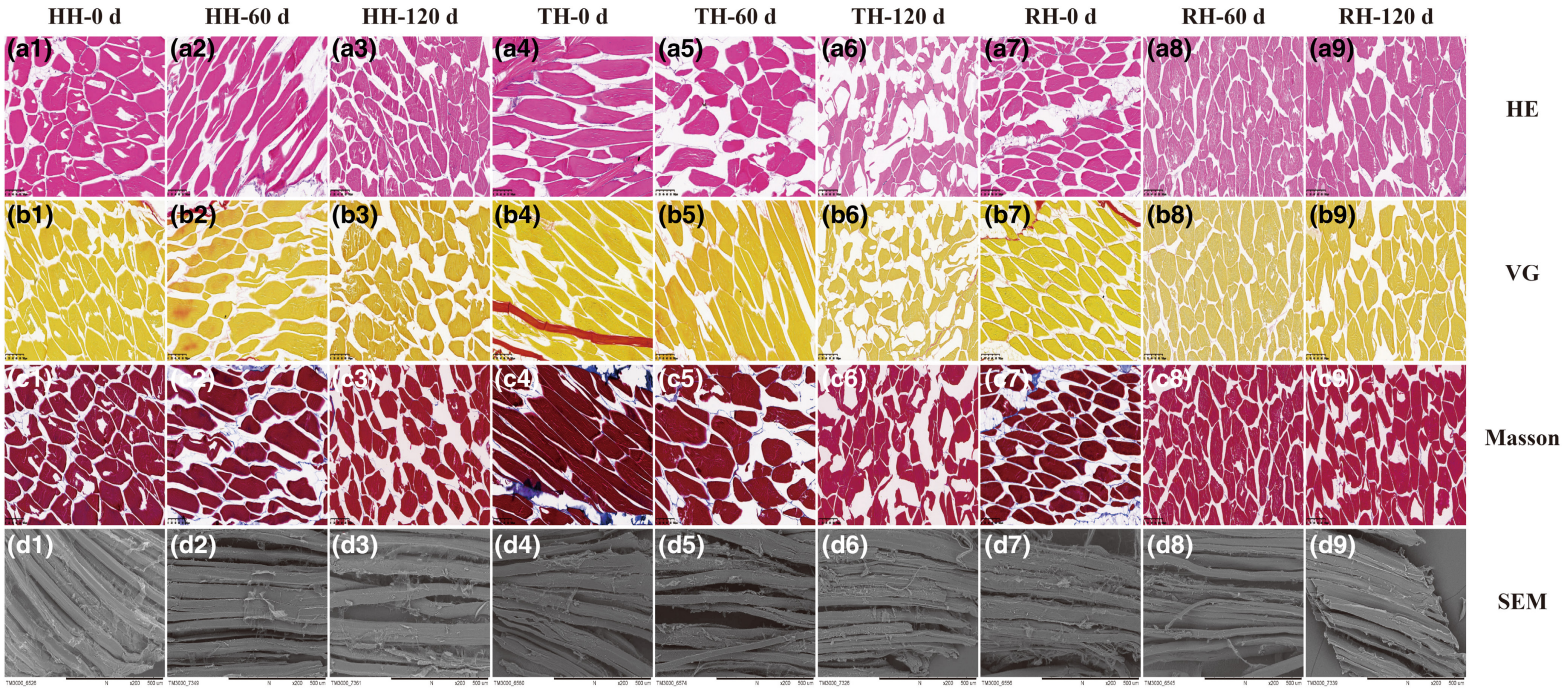
Histological microstructures of hairtail muscle tissues. a1–a9: Hematoxylin and eosin (H&E) staining; b1–b9: Van Gieson (VG) staining; c1–c9: Masson staining; and d1–d9: Scanning electron microscopy (SEM). a1, b1, c1, and d1: Fresh hooked hairtail (HH) muscle (0 d); a2, b2, c2, and d2: HH muscle on 60 d of frozen storage; a3, b3, c3, and d3: HH muscle on 120 d of frozen storage; a4, b4, c4, and d4: Fresh trawl‐net hairtail (TH) muscle (0 d); a5, b5, c5, and d5: TH muscle on 60 d of frozen storage; a6, b6, c6, and d6: TH muscle on 120 d of frozen storage; a7, b7, c7, and d7: Fresh radar‐net hairtail (RH) muscle (0 d); A8, B8, C8, and D8: RH muscle on 60 d of frozen storage; a9, b9, c9, and d9: RH muscle on 120 d of frozen storage.

The histological changes (H&E, VG, Masson staining, and SEM) of HH, TH, and RH muscles on 0, 60, and 120 d of cryopreservation were shown in Figure 4. Observing the figures of H&E staining (Figure 4a1–a9), the hairtail muscle tissues on day 0 exhibited a tight connection of myofibrils (red part) with little white space. However, some myofibrils became incoherent, blurred, and disordered as frozen time extended, which might be attributed to protein denaturation and oxidation under cold stress. In comparison with HH and RH tissues, fresh TH exhibited thicker myofibrils and larger inter‐myofibril spaces. However, myofibrillar fragmentation in TH on days 60 and 120 of storage was more severe, which might be due to the difference in the growth environment, mainly referred to the sea area and depth.

To discriminate collagen fibers from muscle fibers, collagen and myofibrils in HH, TH, and RH tissues were further visualized by VG and Masson staining (Figure 4b1–b9 and 4c1–c9). Collagen fibers were stained red, and myofibrils were yellow in VG staining; and collagen fibers were stained blue, and myofibrils were red in Masson staining. For the fresh fillets, the collagen fibers were tightly interlaced with muscle myofibrils, the interval between myofibrils was small, and the myofibrils were dense. In contrast, freezing treatment, including 60 and 120 days, significantly reduced collagen fibers, widened myofibrillar space, and increased fragmentation. As similarly observed in H&E results, the microscopic structure changes of collagen and muscle fibers in TH samples were the most drastic, matching the textural properties with the lowest chewiness and adhesiveness of TH muscles.

The microstructural changes in the muscle of the hairtail (cross‐section) after being exposed to cold stress were further observed by SEM (Figure 4d1–d9). The muscle bundles of fresh HH, TH, and RH tissues were closely connected, and the space was small, showing a well‐ordered organizational state and structure. After cold cryopreservation, the contraction rate and gap of muscle fibers were obviously enhanced, among which TH samples were the most serious, followed by HH and RH groups, respectively.

The formation and crystallization of ice crystals in muscle tissue have been reported to play enormous implications for maintaining the integrity, strength, and toughness of myofibrils (Zhang et al., 2019). Under cold storage conditions, the growth of ice crystals increases the extracellular space, destroys the myofibril tightness, and weakens the connective tissue and collagen structure (Okuda et al., 2020). These explained the alterations in the structure of the hairtail muscle tissue, which resulted in a reduction in the physical properties of muscle tissue and reduced muscle protein function. Compared with HH and RH muscle tissues, the disruption and tissue fragmentation of TH muscle were most prominent at the end of storage, probably due to the different stability and functional properties of myosin led by different fishing methods and living areas.

### 3.6 Volatile organic compound analysis

**FIGURE 5:**
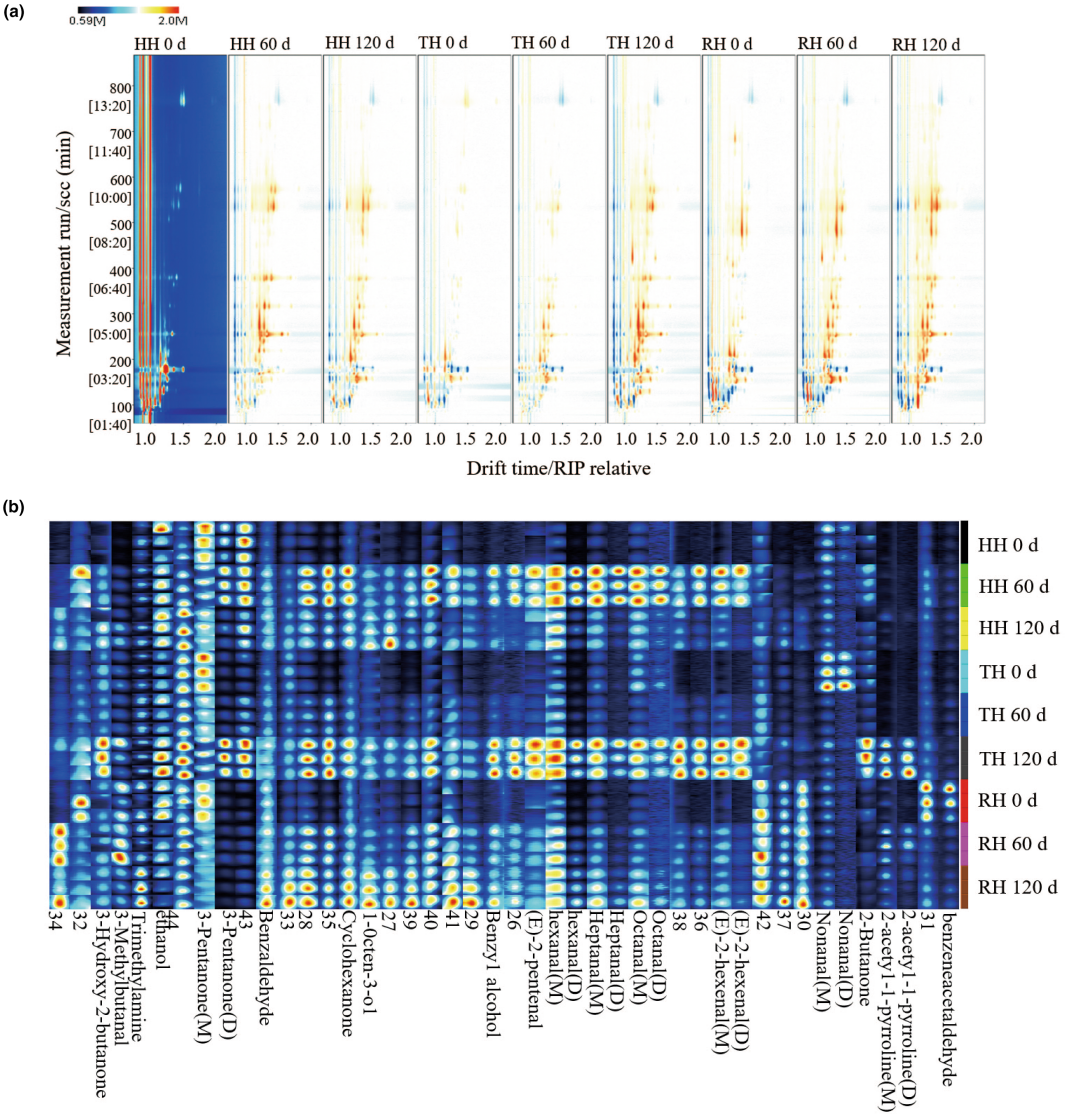
Topographical plot (a) and fingerprinting plot (b) of volatile organic compounds in hairtail muscle. HH 0 d, TH 0 d, and RH 0 d, fresh hooked hairtail (HH), trawl‐net hairtail (TH), and radar‐net hairtail (RH); HH 60 d, TH 60 d, and RH 60 d, HH, TH, and RH on 60 d of frozen storage; HH 120 d, TH 120 d, and RH 120 d, HH, TH, and RH on 120 d of frozen storage.

The changes of VOCs in HH, TH, and RH fillets stored on days 0, 60, and 120, respectively, were visualized in Figure 5. The spectrogram of fresh HH batch (HH 0 d) was selected as the background, and the spectrograms of other batches were deducted from the background to explore the differences of VOCs in different batches (Figure 5a). In the topographical plots, white dots represented that the concentration of VOCs in the batch was equal to those in the reference, blue dots displayed low concentration, and red dots displayed high concentration. The results showed that a large number of differential signals occurred in the retention index of 100–800 s and the migration time of 1.0–1.5 s. The differences in VOCs between fresh HH and TH muscles were relatively small, while relatively higher differences were found between fresh HH and RH muscles, which meant that the flavor composition of hairtail obtained by different fishing methods might vary greatly. With the increase of cryopreservation time, the composition and content of VOCs in the three different hairtails increased significantly, especially in TH samples, which was attributed to protein denaturation and oxidation, lipids oxidation and hydrolysis, and biochemical reactions of residual bacteria in hairtail muscle tissues during cold condition (Wang et al., 2019).

The specific details of VOCs in hairtail muscles were further presented in the fingerprint (Figure 5a) and a table (Table S2), including 14 aldehydes, 5 ketones, 3 alcohols, 3 amines, and 19 unidentified organic compounds. Aldehydes arise from lipid oxidation, such as unsaturated fatty acids, and Streker degradation, which are key VOCs in aquatic and meat products. In addition, the limitation of aldehydes is comparatively low, which made contribution to the aroma of aquatic products by reacting with other substances (Liao et al., 2023). The Maillard reaction and lipid oxidation produce ketones and alcohols, and the alcohols oxidation and the esters decomposition can also form ketones. However, saturated alcohols have a limited effect on the formation of the aroma due to the high odor threshold (Zhan et al., 2022). Amines are mainly generated from the amino acid degradation of high‐protein aquatic products through the action of endogenous protease and lipase, and microbial enzymes, which is often for the purpose of evaluating the freshness of aquatic products (Li et al., 2023).

Compared with the fresh hairtail batch (0 d), the levels of 1‐octen‐3‐ol and benzaldehyde dramatically increased in all hairtail alongside the frozen storage (60 and 120 d). The alcohol 1‐octen‐3‐ol is derived from the degradation of oleic acid, linoleic acid, or palmitoleic acid, and gives off a similar mushroom and earthy taste, which can reflect the levels of lipid oxidation in aquatic products (Liao et al., 2023). The same results of increased 1‐octen‐3‐ol content under cold stress were also found in European seabass (*Dicentrarchus labrax*), Atlantic salmon (*Salmo salar*), and pike‐eel (Muraenesox cinereus) (Du et al., 2022; Kritikos et al., 2020). Benzaldehyde results from the Strecker reaction of phenylalanine and has aromas of cherry, almonds, fruit, cream, and nuts, an increase of which was also presented in air‐dried and fried hairtail fillets (Ding et al., 2022; Liao et al., 2023).

In comparison to HH and TH samples, the content of 2‐acetyl‐1‐pyrroline in RH samples was the highest on days 0 and 60 of frozen storage, while the level of trimethylamine was the highest on days 120. 2‐Acetyl‐1‐pyrroline has been reported as an important contributor to the aroma of cereals, honey, fruit, chocolate, dairy products, cooked meat, and crab, which is described as having nutty, sweet, popcorn, and toasty flavor (Routray & Rayaguru, 2018). The trimethylamine results from the post‐mortem degradation of trimethylamine oxide, which produces an unpleasant fishy odor, thus leading to lower‐quality aquatic products (Liao et al., 2023). These results revealed that fresh RH fillets had an attractive flavor, but the quality was difficult to maintain after a long‐frozen storage period. Additionally, the contents of 2‐butanone, (E)‐2‐pentenal, (E)‐2‐hexenal, 2‐acetyl‐1‐pyrroline, heptanal, octanal, 3‐hydroxy‐2‐butanone, and 3‐pentanone in TH samples at the end of storage were higher than those in other two groups, suggesting the notable deterioration of TH muscle tissues, which was consistent with the above textural characteristics and histological results.

### 3.7 Correlation analysis between physiochemical indexes and VOCs

**FIGURE 6:**
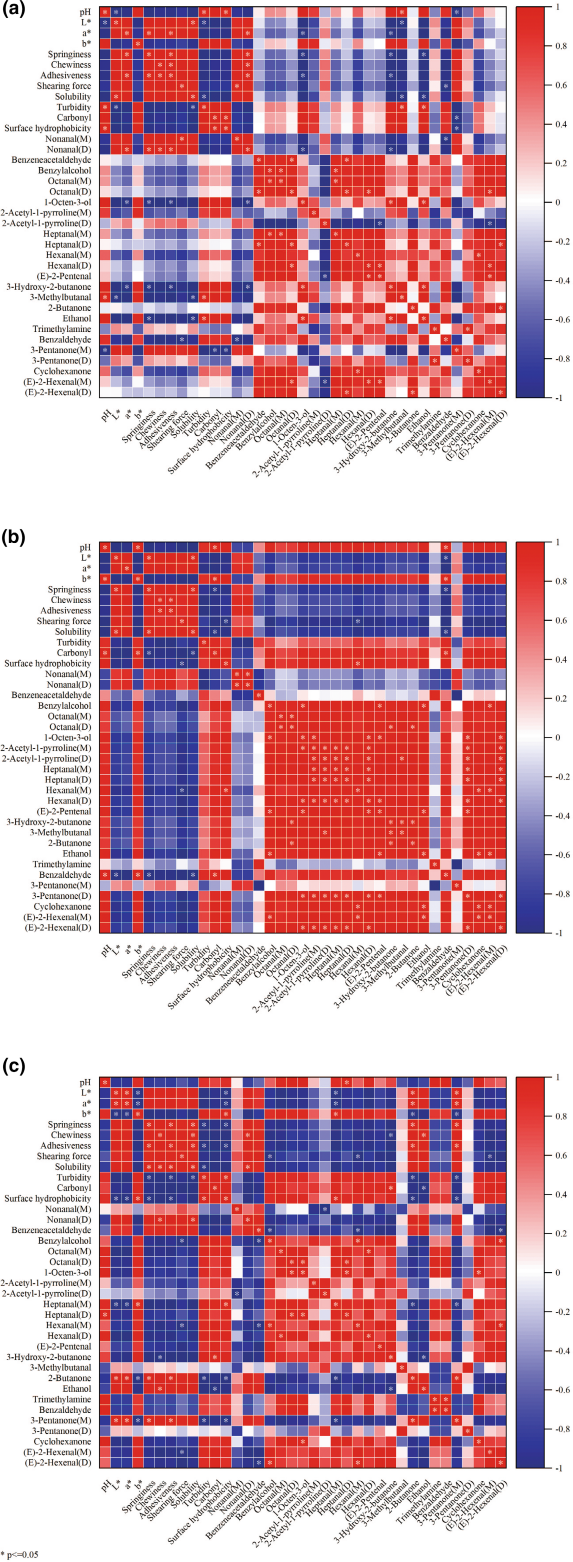
Correlation analysis between physiochemical indexes and volatile organic compounds of hooked hairtail (a), trawl‐net hairtail (b), and radar‐net hairtail (c).

The correlation between physiochemical indicators and VOCs was conducted using Pearson correlation coefficient analysis, as shown in Figure 6. Red represented a positive correlation, blue represented a negative correlation, and asterisks represented a significant correlation (*p* < .05). In the HH sample (Figure 6a), color and texture were positively correlated with nonanal(D) while negatively correlated with 1‐octen‐3‐ol and 3‐hydroxy‐2‐butanone. Protein oxidation was positively correlated with 3‐methylbutanal and ethanol but negatively correlated with 3‐pentanone(M). As for the TH sample (Figure 6b), hexenal(M) and benzaldehyde were positively correlated with protein oxidation. In the RH fillet (Figure 6c), 2‐butanone and 3‐pentanone(M) were positively correlated with color and texture while negatively correlated with protein oxidation. The results revealed that the color, texture, and protein oxidation exhibited close correlations with VOCs during 120 d of frozen storage. It could be because the development of auto‐oxidation and degradation of proteins and lipids led to the deterioration of color and textural properties in hairtails during frozen storage, resulting in variations of VOCs (Du et al., 2022).

## 4 CONCLUSION

In the current study, chemical, histological, and GC‐IMS‐based volatile flavor compound analyses were conducted to explore and compare the quality characteristics of HH, TH, and RH muscles during long‐term cryopreservation. The cold stress led to the increase of pH, b* values, myosin turbidity, carbonyl content, and surface hydrophobicity, and the decrease of L*, a* values, textural properties, myosin solubility in all hairtail batches, indicating the appearance of protein denaturation and oxidation. Moreover, the frozen hairtail samples presented a significantly disordered microstructure with reduced collagen fibers, numerous cracked myofibrils, and intensified fragmentation. In addition, a total of 44 VOCs were identified in three kinds of hairtail, including 14 aldehydes, 5 ketones, 3 alcohols, 3 amines, and 19 unidentified organic compounds. The intensities of 1‐octen‐3‐ol and benzaldehyde in all hairtail markedly increased after freezing, and these two VOCs could be used as biomarkers of frozen hairtail. Specifically, 2‐acetyl‐1‐pyrroline was a key contributor to the aroma of fresh RH fillets. Furthermore, the color, texture, and protein oxidation were closely related to VOCs during 120 d of frozen storage.

HH was fished with a hook and line in the deep ocean offshore, TH was fished with a trawl net in the epipelagic ocean of open seas, and RH was fished with a radar net in the deep ocean offshore. The first capture method was gentler than the latter two but needed to be more efficient and yield less. Compared with HH and RH samples, the texture, microstructure, and flavor of frozen TH muscle tissue were prone to deterioration, indicating poor storage stability of the TH sample. Moreover, fresh RH samples exhibited an attractive aroma with high economic value. This work provides a theoretical basic database for the variation of muscle quality characteristics in different kinds of hairtail during cold conditions. However, the results obtained in this study are still insufficient, and the mechanism of the differences in protein and lipid changes in different hairtail muscles needs further clarification.

## AUTHOR CONTRIBUTIONS

**Shanshan Shui:** Conceptualization (equal); formal analysis (equal); funding acquisition (equal); investigation (equal); methodology (equal); writing – original draft (equal). **Yu Chen:** Formal analysis (equal); investigation (equal). **Hongbo Yan:** Data curation (equal); software (equal). **Jia Song:** Methodology (equal); validation (equal). **Shucheng Liu:** Data curation (equal); investigation (equal). **Soottawat Benjakul:** Conceptualization (equal); supervision (equal). **Bin Zhang:** Conceptualization (equal); funding acquisition (equal); supervision (equal); writing – review and editing (equal).

## CONFLICT OF INTEREST STATEMENT

All authors have no conflicts of interest to declare.

## Supporting information


**Tables S1** **–S2**

## Data Availability

Data will be available on request from the corresponding author.
