# Supplementary material for: Comparative analyses of physicochemical and volatile flavor characteristics in hooked, trawl‐net, and radar‐net hairtail (Trichiurus haumela) muscles during long‐term cryopreservation at −18°C
Source: Food Sci Nutr. 2024 Aug 23;12(10):8159–70. doi: 10.1002/fsn3.4381 (PMC11521657; doi:10.1002/fsn3.4381)
Supplement: Supplementary file 1 — Tables S1 –S2 [file FSN3-12-8159-s001.docx]

Table S1. P values of the different variables measured in hooked hairtail (HH), trawl-net hairtail (TH), and radar-net hairtail (RH) muscles during frozen storage.

| **Storage time** | **Formula** | **pH** | ***L**** | ***a**** | ***b**** | **Springiness** | **Chewiness** | **Adhesiveness** | **Shearing force** | **Solubility** | **Turbidity** | **Carbonyl** | **Surface hydrophobicity** |
| --- | --- | --- | --- | --- | --- | --- | --- | --- | --- | --- | --- | --- | --- |
| **0 d** | **HH** | Ac | Fc | Ea | Ab | Gb | Gc | Gc | Fa | Eb | Aa | Ab | Ac |
|  | **TH** | Aa | Fb | Ec | Aa | Ga | Ea | Ea | Fa | Eab | Aa | Ab | Ab |
|  | **RH** | Ab | Fa | Eb | Ac | Fb | Fb | Fb | Eb | Ea | Ab | Aa | Aa |
| **20 d** | **HH** | Bc | Fc | Da | ABa | Fb | Fb | Fb | Eb | Db | Ba | Ba | Ba |
|  | **TH** | Ba | Eb | Eb | Ba | Fa | Da | Da | Ec | Dab | Bb | Bb | Ba |
|  | **RH** | ABb | EFa | Da | Bb | Eb | Ec | Eb | Da | Da | Bc | Bab | Ba |
| **40 d** | **HH** | BCa | Ec | Da | ABa | Eb | Eb | Ec | Da | Cb | Ca | Ca | Cb |
|  | **TH** | Ca | Db | Db | Ca | Ea | Ca | Da | Db | CDb | Bb | Cb | Ca |
|  | **RH** | ABb | DEa | Ca | BCb | Db | Db | Db | Ca | Ca | Bc | Cb | Ca |
| **60 d** | **HH** | CDb | Dc | Ca | BCa | Db | Db | Db | Ca | Cb | Da | Da | Cb |
|  | **TH** | CDa | Db | Cab | Ca | Da | Ca | Ca | Db | BCb | Cb | Da | Ca |
|  | **RH** | ABCa | CDa | Cb | Cb | Cb | Cb | Cb | Ba | Ba | Cc | Db | Db |
| **80 d** | **HH** | Db | Cc | BCa | Ca | Cb | Cb | Cb | Ba | Bb | Eb | Eb | Db |
|  | **TH** | Da | Cb | Ba | Db | Ca | Ba | Ca | Cc | Bc | CDa | Ea | Da |
|  | **RH** | BCa | BCa | Ba | Dc | Cb | Cb | Ba | Bb | Aa | CDc | Eab | Ec |
| **100 d** | **HH** | Dc | Bc | Ba | Da | Bb | Bb | Bb | Ba | Ab | Eb | Fb | Eb |
|  | **TH** | Eb | Bb | Ba | Ea | Ba | Aa | Ba | Bb | Ac | DEa | Fa | Ea |
|  | **RH** | Ca | ABa | ABa | DEb | Bb | Bc | ABb | ABa | Aa | DEc | EFa | Fc |
| **120 d** | **HH** | Eb | Ab | Aa | Da | Ab | Ab | Aa | Aa | Aa | Fb | Gc | Fb |
|  | **TH** | Ea | Ab | Aab | Fb | Aa | Aa | Aa | Aab | Aa | Ea | Gb | Fa |
|  | **RH** | Da | Aa | Ab | Eb | Ab | Ac | Ab | Ab | Aa | Eb | Fa | Gb |

Different capital letters denote significant differences (*p* < 0.05) as a result of the frozen storage time (0, 20, 40, 60, 80, 100, and 120 d) under the same hairtail specie ; different lower-case letters denote significant differences (*p* < 0.05) as a result of hairtail species (HH, TH, and RH) under the same storage time.

Table S2. The volatile organic compounds (identified compounds) in hooked hairtail (HH), trawl-net hairtail (TH), and radar-net hairtail (RH) muscles during frozen storage.

| **Compound** | **Formula** | **Peak Intensity** | | | | | | | | |
| --- | --- | --- | --- | --- | --- | --- | --- | --- | --- | --- |
|  |  | **0 d** | | | **60 d** | | | **120 d** | | |
|  |  | **HH** | **TH** | **RH** | **HH** | **TH** | **RH** | **HH** | **TH** | **RH** |
| Nonanal(M) | C_9_H_18_O | 1033±76^Ba^ | 1561±132^Aa^ | 697±33^Ca^ | 707±25^Ab^ | 498±29^Bb^ | 509±80^Bb^ | 648±47^Ab^ | 618±85^Ab^ | 635±58^Aab^ |
| Nonanal(D) | C_9_H_18_O | 114±12^Ba^ | 230±31^Aa^ | 88±17^Ba^ | 85±4^Ab^ | 75±10^Ab^ | 79±8^Aa^ | 70±3^Ab^ | 82±9^Ab^ | 75±9^Aa^ |
| Benzeneacetaldehyde | C_8_H_8_O | 178±8^Ba^ | 162±14^Ba^ | 854±251^Aa^ | 199±15^Aa^ | 198±18^Aa^ | 227±20^Ab^ | 181±27^Aa^ | 180±13^Aa^ | 255±52^Ab^ |
| Benzylalcohol | C_7_H_8_O | 100±8^Bc^ | 117±2^Bc^ | 162±10^Ab^ | 340±46^Aa^ | 167±10^Bb^ | 343±12^Aa^ | 215±28^Cb^ | 448±15^Aa^ | 346±62^Ba^ |
| Octanal(M) | C_8_H_16_O | 202±22^Bc^ | 353±37^Ab^ | 212±15^Bc^ | 668±50^Aa^ | 252±6^Cc^ | 336±21^Bb^ | 409±9^Bb^ | 607±41^Aa^ | 521±58^ABa^ |
| Octanal(D) | C_8_H_16_O | 38±1^Bc^ | 56±7^Ab^ | 37±5^Bb^ | 127±7^Aa^ | 41±4^Bb^ | 44±3^Bb^ | 55±5^Bb^ | 104±14^Aa^ | 70±11^Ba^ |
| 1-Octen-3-ol | C_8_H_16_O | 222±19^Bc^ | 271±13^ABb^ | 332±31^Ab^ | 408±8^Ab^ | 290±7^Bb^ | 411±26^Ab^ | 530±36^Ba^ | 476±25^Ba^ | 693±58^Aa^ |
| 2-Acetyl-1-pyrroline(M) | C_6_H_9_NO | 301±18^Ba^ | 274±20^Bb^ | 547±66^Ab^ | 258±30^Ba^ | 287±20^Bb^ | 1340±116^Aa^ | 476±196^Ca^ | 2019±277^Aa^ | 1036±190^Ba^ |
| 2-Acetyl-1-pyrroline(D) | C_6_H_9_NO | 39±5^Aa^ | 38±6^Ab^ | 41±9^Ab^ | 36±2^Ba^ | 30±2^Bb^ | 147±33^Aa^ | 38±4^Ba^ | 302±68^Aa^ | 69±23^Bb^ |
| Heptanal(M) | C_7_H_14_O | 117±13^Bc^ | 198±16^Ab^ | 136±10^Bc^ | 613±25^Aa^ | 194±12^Cb^ | 273±16^Bb^ | 323±9^Cb^ | 534±34^Aa^ | 411±35^Ba^ |
| Heptanal(D) | C_7_H_14_O | 32±3^Ab^ | 35±1^Ab^ | 34±1^Ab^ | 209±23^Aa^ | 34±7^Bb^ | 42±5^Bb^ | 52±1^Bb^ | 147±31^Aa^ | 83±20^Ba^ |
| Hexanal(M) | C_6_H_12_O | 209±14^Bc^ | 293±31^Ac^ | 319±43^Ab^ | 733±15^Aa^ | 419±32^Cb^ | 585±9^Ba^ | 561±14^Bb^ | 756±19^Aa^ | 589±10^Ba^ |
| Hexanal(D) | C_6_H_12_O | 38±5^Bc^ | 59±8^ABb^ | 61±12^Ac^ | 755±64^Aa^ | 90±18^Cb^ | 222±31^Bb^ | 201±33^Bb^ | 614±99^Aa^ | 449±63^Aa^ |
| (E)-2-Pentenal | C_5_H_8_O | 26±3^Ab^ | 22±1^Ac^ | 26±6^Ab^ | 89±14^Aa^ | 32±5^Bb^ | 46±1^Ba^ | 44±5^Bb^ | 104±3^Aa^ | 43±6^Ba^ |
| 3-Hydroxy-2-butanone | C_4_H_8_O_2_ | 104±13^Cb^ | 230±22^Ab^ | 171±30^Ba^ | 208±14^Aa^ | 186±18^Ab^ | 211±22^Aa^ | 278±55^Ba^ | 441±23^Aa^ | 223±17^Ba^ |
| 3-Methylbutanal | C_5_H_10_O | 44±10^Cb^ | 329±47^Ba^ | 707±179^Aab^ | 212±7^Bab^ | 303±8^Ba^ | 1052±254^Aa^ | 394±141^Aa^ | 555±218^Aa^ | 521±38^Ab^ |
| 2-Butanone | C_4_H_8_O | 188±79^Ab^ | 279±12^Ab^ | 213±59^Aa^ | 427±27^Aa^ | 171±5^Bb^ | 160±41^Ba^ | 184±59^Bb^ | 720±103^Aa^ | 117±6^Ba^ |
| Ethanol | C_2_H_6_O | 414±143^Aa^ | 286±26^Ab^ | 498±33^Aa^ | 466±29^Aa^ | 340±60^ABb^ | 312±62^Bb^ | 507±27^Aa^ | 573±81^Aa^ | 255±76^Bb^ |
| Trimethylamine | C_3_H_9_N | 6636±1717^Aa^ | 5762±736^Ab^ | 9943±3077^Ab^ | 7134±121^Aa^ | 9721±326^Aa^ | 9437±2244^Ab^ | 6290±1941^Ba^ | 6693±130^Bb^ | 19038±1625^Aa^ |
| Benzaldehyde | C_7_H_6_O | 170±3^Bb^ | 200±20^Bb^ | 253±14^Ab^ | 242±5^Aa^ | 225±12^Aab^ | 247±19^Ab^ | 252±19^Ba^ | 251±18^Ba^ | 365±23^Aa^ |
| 3-Pentanone(M) | C_5_H_10_O | 282±31^Aa^ | 303±26^Aa^ | 245±10^Aa^ | 242±10^Aa^ | 183±20^Bb^ | 171±21^Bb^ | 178±7^Bb^ | 259±12^Aa^ | 110±3^Cc^ |
| 3-Pentanone(D) | C_5_H_10_O | 1410±226^Ab^ | 420±56^Bb^ | 352±40^Bb^ | 2120±66^Aa^ | 536±19^Bb^ | 503±92^Ba^ | 774±107^Bc^ | 2468±247^Aa^ | 301±16^Cb^ |
| Cyclohexanone | C_6_H_10_O | 186±17^Bc^ | 203±23^Bb^ | 269±6^Ab^ | 482±31^Aa^ | 257±16^Cb^ | 328±19^Bb^ | 372±21^Bb^ | 439±35^ABa^ | 472±46^Aa^ |
| (E)-2-Hexenal(M) | C_6_H_10_O | 65±5^Ac^ | 69±8^Ac^ | 73±8^Ab^ | 267±26^Aa^ | 108±6^Cb^ | 164±8^Ba^ | 118±4^Cb^ | 255±4^Aa^ | 177±20^Ba^ |
| (E)-2-Hexenal(D) | C_6_H_10_O | 14±0^Ab^ | 11±1^Bb^ | 13±2^ABb^ | 81±17^Aa^ | 12±1^Bb^ | 25±3^Ba^ | 17±1^Bb^ | 104±2^Aa^ | 25±6^Ba^ |

Values with different lower-case letters in the same row denote significant differences (*p* < 0.05) as a result of the frozen storage time (0, 60, and 120 d); different capital letters in the same row denote significant differences (*p* < 0.05) as a result of hairtail species (HH, TH, and RH).
